# Impaired hepatic amyloid-beta degradation in Alzheimer’s disease

**DOI:** 10.1371/journal.pone.0203659

**Published:** 2018-09-07

**Authors:** Chera L. Maarouf, Jessica E. Walker, Lucia I. Sue, Brittany N. Dugger, Thomas G. Beach, Geidy E. Serrano

**Affiliations:** 1 Banner Sun Health Research Institute, Sun City, AZ, United States of America; 2 Department of Pathology and Laboratory Medicine, University of California Davis School of Medicine, Sacramento, CA, United States of America; Torrey Pines Institute for Molecular Studies, UNITED STATES

## Abstract

Extensive research strongly suggests that amyloid beta (Aβ) aggregates in the brain have a central role in Alzheimer’s disease (AD) pathogenesis. Pathological Aβ deposition is likely due to an altered balance between overproduction and elimination. Rodent studies have suggested that the liver has a major role in Aβ degradation. It is possible alterations of liver function could affect brain Aβ levels through changes in blood Aβ concentration. In this study, we hypothesized hepatic Aβ degradation to be impaired in AD subjects. To test our hypothesis, an Aβ degradation assay was developed using synthetic fluorescein-labeled Aβ40 and Aβ42 spiked into human liver homogenates. Aβ degradation rates were lower in AD-derived homogenates as compared with those from non-demented (ND) control subjects, even after accounting for such covariates as age, sex, and APOE genotype. The protein expression of potential Aβ-degrading enzymes were also examined. Neprilysin levels were not different in AD liver samples, while cathepsin D and insulin-degrading enzyme were significantly altered in AD subjects. The results support the possibility that impaired hepatic Aβ degradation could be a factor contributing to increased brain Aβ accumulation and AD.

## Introduction

It is well accepted that aggregation of amyloid beta (Aβ) peptides into amyloid plaques is a critical step in the pathogenesis of Alzheimer disease (AD). Studies of early-onset forms of familial AD, Down syndrome, and transgenic rodent models that overexpress normal or mutated forms of the amyloid precursor protein (APP) suggest formation of amyloid plaques may play a key role in the disease [1, 2]. However, it is important to emphasize that familial mutations in humans account for less than 1% of AD cases, and while much of the investigative focus has been on overproduction of Aβ, it is possible that disease initiation or acceleration could also be due to decreased brain clearance or degradation. The major proposed mechanisms of cerebral Aβ elimination are receptor-mediated transport across the blood-brain-barrier and proteolytic degradation in the brain by enzymes such as insulin degrading enzyme (IDE), cathepsin D and neprilysin [3–7]. Experimental animal studies have also indicated that circulating Aβ is metabolized by both liver and kidney [8, 9]. However, to our knowledge, there are no previous studies exploring the ability of the human liver to degrade Aβ and whether this might differ in subjects with and without AD. In this study we used human postmortem liver homogenates to compare Aβ degradation rates in non-demented (ND) control subjects and subjects with AD.

## Materials and methods

### Human subjects

Liver samples came from subjects who were volunteers in the Arizona Study of Aging and Neurodegenerative Disorders (AZSAND), a longitudinal clinicopathological study of normal aging, cognition and movement in the elderly since 1996 in Sun City, Arizona [10]. Autopsies are performed by the Banner Sun Health Research Institute Brain and Body Donation Program (BBDP: www.brainandbodydonationprogram.org). All subjects signed Western Institutional Review Board-approved informed consents allowing both clinical assessments during life and several options for brain and/or bodily organ donation after death." The name of the IRB is Western Institutional Review Board, Seattle Washington and its approval include the collection of human organs at autopsy for unlimited number of research studies, including this one. Most subjects are clinically characterized with annual standardized test batteries consisting of general neurological, cognitive and movement disorders components, including the Mini Mental State Examination (MMSE). Subjects for the current study have had a complete pathological evaluation by a licensed pathologist (**Table 1**; N = 16) and were chosen by searching the BBDP database for cases with a whole-body autopsy and a clinicopathological diagnosis of AD (N = 8) or control (N = 8) and low post mortem interval. Exclusion criteria included clinical history of liver disease, hepatitis, severe fibrosis and cirrhosis, as well as other comorbid brain pathology including Lewy body disease, vascular dementia and non-AD tauopathies.

**Table 1:** Patient demographics.

Diagnosis	Expired Age, years (SD)	Gender	PMI, hours (SD)	Total Plaque Score (SD)	Liver Weight (SD)
NDC (n = 8)	83 (8.7)	8M:0F	2.9 (0.48)	4.25 (4.92)	1431.3g (457.7)
AD (n = 8)	83 (7.7)	5M:3F	3.0 (0.54)	14.38 (0.88)*	1064.4g (305.9)

NDC, non-demented control; AD, Alzheimer’s disease; SD, standard deviation; M, male; F, female; PMI, postmortem interval

* = p<0.0001

### Pathological examination

Complete pathological examination was performed using standard AZSAND methods [10, 11] and consisted of gross and microscopic examination, including pathologist assessment of both brain and peripheral organs. All areas were stained with hematoxylin and eosin for general pathological assessment. Standard brain areas were also stained with thioflavin S, Gallyas, and Campbell-Switzer methods to detect the presence of senile plaques, neurofibrillary changes and other neuronal and glial tauopathies [10–14]. Immunohistochemical staining was used to document the presence of alpha-synuclein pathology [15–17]. Neuritic plaque and neurofibrillary tangle (NFT) densities were graded blindly as recommended by CERAD with separate semi-quantitative density estimates of none, sparse, moderate, or frequent [18]; all scores were converted to a 0–3 scale for statistical purposes. Regions scored included cortical gray matter from frontal (F), temporal (T), parietal (P), hippocampal CA1 (H), and entorhinal (E) regions. Neurofibrillary degeneration was also staged on thick frozen sections by the original method of Braak [12], and clinicopathological AD diagnoses were made when subjects were demented and met “intermediate” or “high” probabilities, according to National Institute on Aging/Reagan Institute criteria [19–21].

### Liver homogenization

Frozen liver (~500 mg) was ground into a powder in liquid nitrogen with a mortar and pestle then transferred to 8 ml sucrose lysis buffer (SLB: 20 mM HEPES, 1.5 mM magnesium chloride, 10 mM potassium chloride, 40 mM sucrose, 1 mM EDTA, 2% glycerol, 0.5% sodium deoxycholate, 1% Tergitol, pH 7.9) at 4°C. The tissue was then homogenized using an Omni TH tissue grinder (Kennesaw, GA) at 4°C. The homogenates were centrifuged at 15,000 x g in a Type 50.4 Ti rotor (Beckman Coulter, Brea, CA) for 20 min at 4°C and the supernatant was collected. Total protein was determined with Pierce’s Micro BCA protein assay kit (Thermo Fisher Scientific, Waltham, MA).

### Degradation of fluorescein-labeled Aβ

Lyophilized fluorescein-labeled Aβ40 and Aβ42 peptides (FAβ, rPeptide, Bogart, GA) were reconstituted in DMSO to 1 mg/ml and stored at 4°C [22]. For degradation experiments, the stock peptides were diluted to 0.4 μg/ml (1:2500) in sucrose lysis buffer (SLB), 5 μl of either FAβ40 or FAβ42 was added to the reaction mixtures and samples were incubated at 37°C for 1 h or 2 h (for ELISA and Western experiments). In addition, two 0 h incubations were included: one liver homogenate with 5 μl of FAβ as well as a negative control sample without FAβ. Reactions were terminated by placing homogenates on ice and by adding SLB with complete protease inhibitor cocktail (PIC, Roche, Mannheim, Germany). Reactions used for Western blots were stopped by 2XLDS sample buffer (Invitrogen) containing 100 mM dithiothreitol.

### Analysis by ELISA and immunoblot

All 4 sample sets were analyzed in triplicate with Aβ40 and Aβ42 ELISAs (Invitrogen; catalog **#** KHB3481 and KHB3441 following the manufacturer’s instructions. Similar sets of samples were analyzed for FAβ by Western blot (as described below) with an anti-fluorescein antibody **(Table 2)** which binds to both to both free and bound fluorescein.

Liver homogenates were assessed for Aβ-degrading enzymes as follows: Eight μg of total protein was brought up to 20 μl with NuPage 2XLDS sample buffer (Invitrogen, Carlsbad, CA) containing 100 mM dithiothreitol then heated at 80°C for 10 min. The liver proteins were separated on 4–12% Bis-Tris gels (Invitrogen; catalog # NP0336BOX) using NuPage 1XMES run buffer and NuPage antioxidant (Invitrogen). The Precison Plus Protein Dual Xtra Prestained Protein Standard from Bio-Rad (Hercules, CA; catalog # 161–0377) was used to determine molecular weight. NuPage transfer buffer with 20% methanol was used to transfer proteins onto 0.45 μm pore nitrocellulose membranes. The membranes were blocked with 5% Quick-Blocker (G-Biosciences, St. Louis, MO) in phosphate buffered saline (PBS), 0.5% Tween 20 (PBS-T), then incubated overnight at 4°C in primary diluted in blocking buffer (see **Table 2** for list of antibodies). The membranes were washed 4X in PBS-T for 5 min each and the blots were then incubated in secondary antibody **(Table 2)** that was also diluted in blocking buffer for 1 h at room temperature. SuperSignal WestPico Chemiluminescent substrate (Thermo Fisher Scientific), CL-Xposure film and a Konica Minolta SRX-101A film processor (Wayne, NJ) were used to detect protein bands. A GS-800 calibrated densitometer (Bio-Rad) scanned the films and Quantity One software (Bio-Rad) was used for densitometry analysis.

**Table 2:** Antibodies used in western blots.

Primary antibody	Antigen specificity or immunogen	Secondary antibody	Company/Catalog #
Anti-fluorescein	KLH-bound fluorescein	M	Sigma Aldrich/11426320001
Neprilysin (anti-CD10)	Synthetic peptide corresponding to Human CD10 aa 50–500	R	Abcam/ab126593
Cathepsin D	Human cathepsin D	M	BD Transduction Labs/610800
IDE	purified human erythrocyte IDE	M	Covance/mms-282R
GAPDH	Full-length human GAPDH protein	M	Life Technologies/39-8600

aa, amino acid; CTF, C-terminal fragment; IDE, insulin degrading enzyme; GAPDH, glyceraldehyde 3-phosphate dehydrogenase; M, HRP conjugated AffiniPure goat-anti mouse IgG (catalog # 115-035-146, Jackson Laboratory); R, HRP conjugated AffiniPure goat-anti rabbit IgG, (catalog # 111-035-144, Jackson Laboratory).

### Statistical methods

For comparing group measures, the Mann-Whitney U-Test, were used to analyze group differences, linear and Spearman regressions were used to correlate % Aβ degradation with post-mortem interval (PMI), number of liver pathologies, age at death and liver weight. Multiple linear regression was applied to study possible correlation between the observed benign liver pathology and hepatic Aβ degradation.

## Results

Both groups had similar ages at death and PMI (Table 1). Half of the cases in each group were heterozygous for the APOE allele 4, while the other half were non-carriers. Some cases had miscellaneous benign hepatic pathology such as liver atrophy, passive liver congestion and mild steatohepatitis, but the proportion of these were not different between the ND and AD group (S1 Table). There was a trend for liver weight loss in AD subjects, but this difference was not statistically significantly different. Multiple linear regressions and Spearman correlations were used to analyze possible correlations of hepatic Aβ degradation with benign liver pathologies, age, PMI and liver weight, but no significance was found with any of the variables analyzed.

**Fig 1:**
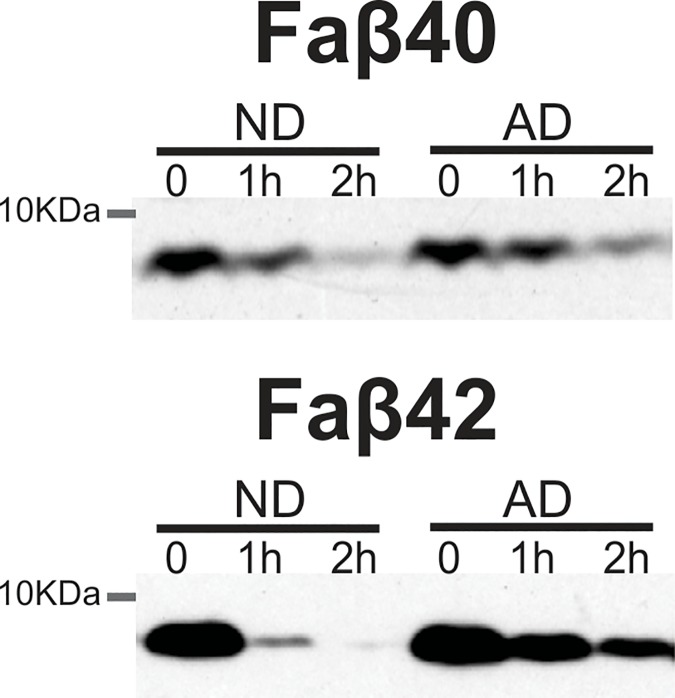
Representative western blot showing faster Aβ degradation in the liver of non-demented controls subjects when compared to the AD group. Lyophilized fluorescein-labeled Aβ40 (A) or Aβ42 (B) peptides were added to liver homogenates to quantify its degradation. Sucrose lysis buffer containing complete protease inhibitor cocktail was added to stop the reaction and Western blot performed to visualized Aβ degradation (molecular weight ~7kDA).

**Fig 2:**
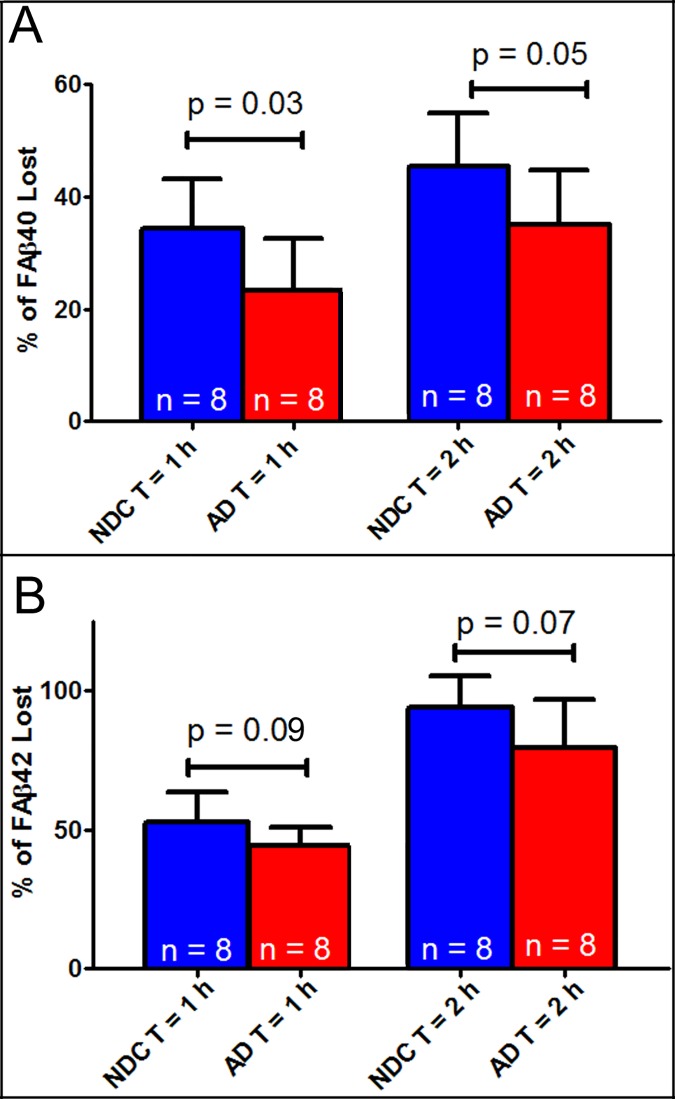
ELISA measurements of Aβ40 and Aβ42 degradation in liver homogenates from non-demented controls (NDC) and Alzheimer’s disease (AD) subjects. Homogenates from NDC degraded Aβ faster than AD subjects. Aβ40 degraded slower (A) than Aβ42 (B) and even though both peptides degraded faster in the NDC group.

**Fig 3:**
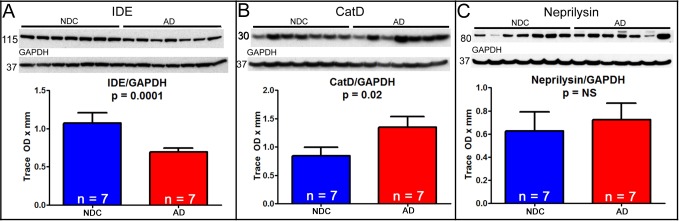
Protein expression of proteolyic degradation enzymes, IDE, CatD and neprilysin in the liver of non-demented controls (NDC) and Alzheimer’s disease (AD) subjects. Liver protein expression of the proteolytic degradation enzyme insulin degrading enzyme (IDE) was lower in AD subjects (A); while cathepsin (CatD) levels (B) were higher. Neprilysin was not statistically significantly between the two groups (C). GAPDH was used as a total protein loading control.

As expected, the CERAD neuritic plaque density and Braak stage showed significant differences when the ND controls were compared to the AD group (**Table 1**; p<0.0001). Western blot (**Fig 1**) and ELISA (**Fig 2**) results showed faster Aβ degradation in the liver of ND subjects as compared to the AD group. Thirty-four percent of Aβ40 was degraded within an hour in the ND group compared to 23% in the AD samples (p<0.05) **(Fig 2)**. Surprisingly, Aβ42 degraded faster than Aβ40. Within 2 hours, 94% of Aβ42 was degraded by the ND homogenates while this was 80% for the AD group (p = 0.07) **(Fig 2)**. Basal concentrations Aβ40 or Aβ42 were not detectable without the presence of the monomeric fluorescein-labeled Aβ40 or Aβ42 peptides. The presence of the APOE 4 allele did not show an effect on Aβ degradation rate. Western blots showed that AD liver homogenates had lower expression of IDE (*p<0*.*001*) and higher levels of cathepsin D (**Fig 3**; *p<0*.*05*) while there was no significant difference in neprilysin levels.

## Discussion

It is well known that neurotoxic Aβ peptides form into insoluble filaments that accumulate into cerebral amyloid plaques and around blood vessel [23, 24]. There is also recognition that amyloid deposition may be triggered by either overproduction or decreased brain elimination of Aβ [7]. It is possible both scenarios may be occurring in the aged brain, along with many other factors that disrupt brain homeostasis and precipitate neurodegeneration. While many studies have focused on Aβ production and clearance within the brain, to our knowledge, there have been no prior investigations of the role of the liver in Aβ metabolism in human subjects. In this study we investigated whether the metabolism of Aβ in the liver is affected in AD using fluorescein-labeled Aβ40 and Aβ42 peptides. The rapid metabolism rates of these peptides in the liver make it almost impossible to relay on endogenous levels of Aβ detected after death, even at short PMI. Therefore, we took advantage of methodologies previously published by M.A. Leissring, et al in 2003 [22], where they demonstrated that monomeric fluorescein-labeled Aβ40 and Aβ42 peptides were very stable and behaved similarly to wildtype Aβ, and investigated the metabolic capabilities of liver homogenates using these peptides. Ghiso et al. 2004 showed in animals that peripheral organs such as the liver, kidney, stomach and spleen are also involved in Aβ clearance. Their results suggest that liver is the main organ that metabolized more than 60% of Aβ [8]. Our results validate the involvement of the liver degrading Aβ in humans and are also indicative of possible Aβ metabolism deficiencies in the liver of AD subjects. The mechanism of such deficiency is unclear, however the expression of two proteolytic enzymes were altered in AD liver as previously observed in AD brain [25, 26]. The concentration of cathepsin D was increased in the AD group while that of insulin degrading enzyme was decreased. Aβ can be cleared through the autophagy-lysosomal system and it is been well reported that deficit in the lysosomal degradation function leads to accumulation of Aβ aggregates [26, 27]. Lysosomal proteases, such as cathepsin D plays a major role in AD through clearance of accumulated Aβ aggregates and previous studies have reported elevated expression in neurons of AD subjects. This apparent discrepancy is not yet clear and to our knowledge have not been well studied. What is well established is that any dysregulation in the protease expressions and/or their proteolytic activities disrupts cellular homeostasis. We speculate that elevation of protein expression could be a compensatory mechanism due to decreased proteolytic activity.

Decreased liver Aβ metabolism might result in brain Aβ accumulation, because one can hypothesize this could result in elevated blood Aβ levels throughout an individual’s life and could result in increased entry of Aβ into the brain. Over decades, disruption of homeostatic Aβ entry and clearance could eventually result in brain plaque formation, analogous to the early onset of Aβ brain deposits in Down’s syndrome due to whole-body overexpression of APP [28, 29]. Many have postulated once plaques form in the brain, they could then act as a “sink”, drawing down blood Aβ concentrations [30, 31]. However, removing Aβ from the periphery with solanezumab does not seem to remove PET-detectable Aβ from the brain, therefore it might seem that modification of peripheral metabolism might be irrelevant to AD. The failure of solanezumab to remove Aβ deposits from the brain could be due to many factors, but perhaps most plausibly because once Aβ is sequestered in insoluble plaques, a simple concentration gradient may not be sufficient to dissolve the deposits. We acknowledge, however, that our findings showing lower levels of hepatic Aβ degradation rates in AD could be a result of the disease, rather than a contributing factor to disease and it is possible liver homogenates in vitro may not faithfully mimic liver metabolism in vivo, but these results are intriguing and support further study of the role of the liver in AD pathogenesis.

## Supporting information

S1 Table Patient hepatic pathologies. Miscellaneous Benning Hepatic Pathologies present in all the cases used in this study.
(DOCX)
