# Supplementary material for: Impaired hepatic amyloid-beta degradation in Alzheimer’s disease
Source: PLoS One. 2018 Sep 7;13(9):e0203659. doi: 10.1371/journal.pone.0203659 (PMC6128628; doi:10.1371/journal.pone.0203659)
Supplement: S1 Table — Miscellaneous Benning Hepatic Pathologies present in all the cases used in this study. (DOCX) [file pone.0203659.s001.docx]

**Supplemental Table 1: Patient Hepatic Pathologies**

| **DX** | **Miscellaneous Benning Hepatic Pathologies** |
| --- | --- |
| ND | Liver organomegaly; acute passive congestion |
| ND | Hepatic atrophy |
| ND | Mild chronic portal hepatitis. |
| ND | Patchy, mild chronic portal hepatitis, etiology undetermined |
| ND | No liver pathology |
| ND | Hepatic atrophy |
| ND | No liver pathology |
| ND | Hepatic atrophy; acute passive congestion |
| AD | Mild portal hepatitis; mild passive congestion; hepatic atrophy |
| AD | Hepatic capsular cyst |
| AD | Mild chronic passive congestion. |
| AD | Mild steatohepatitis |
| AD | Hepatic atrophy |
| AD | No liver pathology |
| AD | No liver pathology |
| AD | Hepatic atrophy |

NDC, non-demented control; AD, Alzheimer’s disease;
